# Incidence of Anxiety and Depression Among Patients with Type 2 Diabetes and the Predicting Factors

**DOI:** 10.7759/cureus.4254

**Published:** 2019-03-14

**Authors:** Paeenda Khan, Neyha Qayyum, Farina Malik, Tooba Khan, Maaz Khan, Amber Tahir

**Affiliations:** 1 Miscellaneous, Jinnah Sindh Medical University, Karachi, PAK; 2 Internal Medicine, Jinnah Postgraduate Medical Centre, Karachi, PAK; 3 Internal Medicine, Dow University of Health Sciences, Karachi, PAK

**Keywords:** hospital anxiety and depression scale, diabetes mellitus, type 2 diabetes mellitus, depression in diabetes, depression, anxiety, pakistan

## Abstract

Introduction

Diabetes mellitus (DM) is a chronic, progressive metabolic illness which is commonly complicated by coexistence of depression and anxiety. This study aimed to assess the prevalence of anxiety and depression among diabetic patients and the factors predicting this coexistence.

Methods

It was a cross-sectional, observational study which included patients of type 2 DM admitted in the hospital due to diabetes-related condition - diabetic foot infections/ulcers, hyperosmotic hyperglycaemic state (HHS), and hypoglycaemic coma/seizure. Anxiety and depression were measured by using the Hospital Anxiety and Depression Scale (HADS). Data was entered and analysed using SPSS version 22.0 (IBM Corp., Armonk, NY, USA).

Results

Mean anxiety score of the participants was 10.88 ± 4.075 and mean depression score was 11.82 ± 4.049. There were 72 (50.7%) patients who had anxiety and 70 (49.2%) patients who had depression. Higher scores of anxiety and depression were statistically significant in female gender, older participants, individuals with longer duration of diabetes, those taking non-insulin treatment, and individuals with painful neuropathy, nephropathy, and foot ulcers.

Conclusion

The incidence of depression and anxiety among hospitalized patients of diabetes mellitus is high. The coexistence of these two chronic debilitating illnesses is worsening the overall quality of life. It is very important to diagnose and manage anxiety and depression in patients with type 2 DM to ensure higher quality of life and life expectancy.

## Introduction

Diabetes mellitus (DM) is among the most common chronic metabolic illnesses and is characterized by elevated plasma glucose levels. Individuals living with DM become more prone to health complications, have an overall reduced quality of life, and result in higher burden on the economy due to their medical care expenditure [1]. In 2017, it was estimated that globally USD 850 billion has been spent on the healthcare of persons living with diabetes [2]. In 2018, there were 500+ million individuals living with type II DM globally. Although currently the prevalence is comparable in high-income and low-income countries; the expected rise in prevalence will be more aggressive in low-income countries [3].

According to World Health Organization (WHO), the global leading cause of disability is depression with 300 million individuals succumbing to this debilitating mental disorder [4]. Epidemiological studies have shown that coexistence of diabetes mellitus and depression is twice as frequent as either of these alone [5].

Diabetes-related risk factors that predispose to development of depression have been studied by many researchers. In people with type 2 DM, greater incidence of depression was seen in insulin-dependent individuals than those who were non-insulin dependent [6]. This implies that insulin-dependent individuals had more progressed disease and increased demand of treatment. As the disease further progresses and complications of diabetes ensue, especially painful peripheral neuropathy and sexual dysfunction, the incidence of depression in the individuals further rises [7]. Among all diabetic complications, neuropathy and nephropathy have been seen to be the strongest predictors of depression [8, 9].

Coexistence of depression and DM becomes a grave challenge for the clinicians as both illnesses worsen the outcome of each other. Combination of depression and DM reduces overall quality of life, impairs self-management of diabetes, increases the risk of diabetic complications, and reduces overall life expectancy [10]. Diabetic patients with coexisting depression have reported higher HbA1c, and more derangement in blood sugar levels [9]. In order to ensure an adequate quality of life, the multidimensional approach to manage diabetes mellitus should also take into consideration the mental health of these individuals and identifying the ones who are at risk of succumbing to the grave illness of depression. The aim of this study is to assess the frequency of depression in diabetic patients admitted in the hospital due to any diabetes-related reason.

## Materials and methods

This observational, cross-sectional study was conducted in the department of general medicine, Jinnah Post Graduate Medical Centre, from October till December 2018. Known cases of type 2 diabetes mellitus, of both genders, who were admitted in the hospital (for minimum one day) for diabetes-related reasons including diabetic foot infections/ulcers, hyperosmotic hyperglycaemic state (HHS), and hypoglycaemic coma/seizure, were invited to participate in the study. Patients of type 1 diabetes, and those type 2 diabetics who had coexisting hypertension or cardiovascular disease were excluded to reduce bias. Hospital Anxiety and Depression Scale (HADS) [11] was utilized to assess the frequency of anxiety and depression in these patients. HADS is a 14-item self-administered instrument that has two subscales to assess anxiety and depression. Each subscale has seven items. Each item is evaluated on a four-point Likert scale. All scores are then added. A combine score of 0-7 indicates “no anxiety/depression,” score of 8-10 indicates “moderate anxiety/depression,” and a score of 11-21 indicates “severe anxiety/depression.”

Along with HADS, a demographic performa was created which included patient gender, age, duration of DM, treatment of DM, complications of DM, duration of hospital admission, and the reason of admission. Data was entered and analysed using SPSS version 22.0 (IBM Corp., Armonk, NY, USA). Mean and standard deviation (SD) was calculated for continuous variables including patient age, duration of hospital stay, and mean scores of depression and anxiety on HADS. Internal consistency of both subscales of HADS was calculated using Cronbach’s alpha. Frequencies and percentages were calculated for categorical variables such as gender, severity of depression and anxiety. Chi square was applied to compare severity of HADS with patient characteristics. p-value of ≤0.05 was considered significant.

## Results

The study was completed by 142 diabetic patients. The mean age of the participants was 57.01 ± 11.23 years (range: 45-71 years). Their mean duration of hospital stay was 3.14 ± 1.58 days (range: 1-7 days). Of all the participants, 61 (43%) were males and 81 (57%) were females. The demographic and clinical characteristics of the patients are shown in Table 1.

**Table 1:** Demographic and clinical characteristics of the patients (n = 142).

Patient characteristics	Frequency n (%)
Gender	
Male	61 (43%)
Female	81 (57%)
Age	
Less than 50 years	39 (27.4%)
More than 50 years	103 (72.5%)
Duration of diabetes	
Less than 10 years	40 (28.2%)
More than 10 years	102 (71.8%)
Treatment of diabetes	
Insulin therapy	48 (33.8%)
Non-insulin medications	94 (66.2%)
Complications of diabetes	
Peripheral painful neuropathy	32 (22.5%)
Nephropathy	17 (11.9%)
Retinopathy	11 (7.7%)
Foot ulcers	48 (33.8%)
Sexual dysfunction	9 (6.3%)
No complication	25 (17.6%)
Reason for admission	
Infected diabetic foot ulcer	43 (30.2%)
Hyperosmotic hyperglycaemic state	52 (36.6%)
Hypoglycaemic coma/seizures	47 (33.1%)

The internal consistency of HADS in this study was 0.78. The internal consistency of subscale anxiety was 0.71 and that of subscale depression was 0.75. Overall mean anxiety score was 10.88 ± 4.075 and mean depression score was 11.82 ± 4.049. There were 72 (50.7%) patients who had anxiety and 70 (49.2%) patients who had depression. The severity of their anxiety and depression on HADS is shown in Table 2.

**Table 2:** Frequency and severity of anxiety and depression on HADS in patients with type 2 diabetes mellitus (n = 142). HADS: Hospital Anxiety and Depression Scale

Subscale of HADS	Frequency n (%)
Anxiety	
HADS score > 10	72 (50.7%)
Mild	35 (48.6%)
Moderate	19 (26.3%)
Severe	18 (25%)
Depression	
HADS score > 10	70 (49.2%)
Mild	16 (22.8%)
Moderate	29 (41.4%)
Severe	25 (35.7%)

HADS scores of both subscales - anxiety and depression - were correlated with the demographic and clinical characteristics of the patients as shown in Table 3. It was seen that anxiety score of more than 10 was more common in female gender, older participants, individuals with longer duration of diabetes, those taking non-insulin treatment, individuals with painful neuropathy and nephropathy, and those admitted with HHS (Table 3).

On the other hand, higher score of depression on HADS was more common in women, older age participants, individuals with longer duration of diabetes, those taking non-insulin treatment, individuals with painful neuropathy and foot ulcers, and individuals admitted with those admitted with foot ulcers (Table 3). Except for age, the scores of HADS were statistically significant to all other demographic and clinical characteristics as shown in Table 3.

**Table 3:** HADS score of anxiety and depression categorized according to the demographic and clinical characteristics of the patients (n = 142). HADS: Hospital Anxiety and Depression Scale

Demographic and clinical characteristics	Anxiety			Depression		
	HADS score > 10 (n = 72)	HADS score < 10 (n = 70)	p-value	HADS score > 10 (n = 70)	HADS score < 10 (n = 72)	p-value
Gender						
Male	22 (30.5%)	39 (55.7%)	0.002	24 (34.2%)	37 (51.4%)	0.03
Female	50 (69.5%)	31 (44.3%)		46 (65.7%)	35 (48.6%)	
Age						
Less than 50 years	18 (25%)	21 (30%)	0.50	22 (31.4%)	17 (23.6%)	0.29
More than 50 years	54 (75%)	49 (70%)		48 (68.5%)	55 (76.3%)	
Duration of diabetes						
Less than 10 years	9 (12.5%)	31 (44.2%)	<0.0000	11 (15.7%)	29 (40.2%)	0.001
More than 10 years	63 (87.5%)	39 (55.7%)		59 (84.3%)	43 (59.8%)	
Treatment of diabetes						
Insulin therapy	29 (40.2%)	19 (27.1%)	0.09	32 (45.7%)	16 (22.2%)	0.003
Non-insulin medications	43 (59.8%)	51 (72.8%)		38 (54.3%)	56 (77.7%)	
Complications of diabetes						
Peripheral painful neuropathy	27 (37.5%)	5 (7.1%)	0.00071	22 (31.4%)	10 (13.9%)	0.008
Nephropathy	9 (12.5%)	8 (11.4%)		6 (8.5%)	11 (15.3%)	
Retinopathy	3 (4.2%)	8 (11.4%)		1 (1.4%)	10 (13.9%)	
Foot ulcers	19 (26.7%)	29 (41.4%)		25 (35.7%)	23 (31.9%)	
Sexual dysfunction	5 (6.9%)	4 (5.7%)		5 (7.1%)	4 (5.5%)	
Reason for admission						
Infected diabetic foot ulcer	26 (36.1%)	17 (24.2%)	0.000	29 (41.4%)	14 (19.4%)	0.01
Hyperosmotic hyperglycaemic state	34 (47.2%)	18 (25.7%)		19 (27.1%)	33 (45.8%)	
Hypoglycaemic coma/seizures	12 (16.6%)	35 (50%)		22 (31.4%)	25 (34.7%)	

## Discussion

Diabetes mellitus is a chronic, progressive, metabolic illness and superimposed depression further debilitates the overall health-related quality of life and life expectancy of these individuals. Almost half of the study participants in this survey were anxious and depressed. The associated factors included female gender, longer duration of diabetes, non-insulin treatment, complications including neuropathy, nephropathy, and foot ulcers, and admission due to infected foot ulcers, HHS, and hypoglycaemic coma.

The outcomes of this study are comparable to those existing in the literature and reinforce the higher incidence of anxiety and depression in type 2 DM patients. In a study conducted in Tunisia, 40% of elderly diabetics were anxious and 22% were depressed [12]. Khuwaja et al. in their study reported the incidence of depression and anxiety to be 44% and 58%, respectively [13]. However, in the western countries the reported incidence of depression and anxiety among diabetics is slightly lower. Collins et al. utilized HADS in their study and reported 32% diabetics to be depressed and 22% anxious [14].

The diabetes-related factors predicting the incidence of depression reported in this study correspond to other existing literature. Painful peripheral diabetic neuropathy has been repeatedly reported to be associated with onset of depression [9, 15]. This association can be well explained by the underlying neurochemical imbalance [16]. Although other studies have reported incidence of depression to be higher in diabetics who are dependent on insulin for their treatment [12, 17], our study reported different outcomes. This may imply that this association might not be valid for Pakistani population or maybe the individuals being managed with oral hypoglycaemic agents need to be switched to insulin therapy for better control and more promising outcomes. Since we could not find any Pakistani study reporting this association, we would recommend larger, multiple studies to strengthen this association. This study is in no way enough to establish that depression is more common in non-insulin dependent individuals.

While this study adds to the limited data available regarding anxiety and depression among hospitalized patients with type 2 DM, it has its limitations too. Since it is a cross-sectional study, a causal relationship between diabetes and symptoms of anxiety and depression cannot be proven or established. Another limitation is that, instead of a structured interview to evaluate anxiety and depression, a psychometric scale was utilized which makes it hard to include physical aspect of psychiatric symptoms. The survey was only conducted among hospitalized patients who might have higher prevalence of anxiety and depression than non-hospitalized diabetic patients.

Despite these limitations, as suggested by this study and supported by the global data, the coexistence of these two chronic debilitating illnesses is worsening the overall quality of life. It is very important to diagnose and manage anxiety and depression in patients with type 2 DM, which is only possible with a collaborative effort of diabetologists, general physicians, and psychiatrists.

## Conclusions

The incidence of depression and anxiety among hospitalized patients of diabetes mellitus is high. Strong predictors of depression in patients with diabetes include female gender, longer duration of diabetes, non-insulin treatment, and complications including neuropathy, nephropathy, and foot ulcers. The coexistence of these two chronic debilitating illnesses is worsening the overall quality of life. It is very important to diagnose and manage anxiety and depression in patients with type 2 DM, which is only possible with a collaborative effort of diabetologists, general physicians, and psychiatrists.
